# Chemical analysis and filtration efficiency of ceramic point-of-use water filters^☆^

**DOI:** 10.1016/j.heliyon.2023.e18343

**Published:** 2023-07-17

**Authors:** Ohene B. Apea, Edem Bennet Akorley, Emmanuel O. Oyelude, Boateng Ampadu

**Affiliations:** a Department of Applied Chemistry, C. K. Tedem University of Technology and Applied Sciences, Ghana; b Department of Environmental Science, C. K. Tedem University of Technology and Applied Sciences, Ghana

**Keywords:** Trace metal, Physicochemical, Water quality index, Environment, Adsorption, Geology

## Abstract

Ceramic water filters (CWFs) are globally employed as a point-of-use water treatment technology. Although, there are no standards to regulate the use of these CWFs in developing countries, they are gaining acceptability for domestic water treatment. This study sought to assess and compare the efficiency of commercially available types of CWFs and to propose a consumer selection guide for the purchase and use of CWFs. The CWFs selected for the study were, Ball filter with activated carbon (BF + AC), Candle filter (CF), and Pot filter (PFcs) coated with colloidal silver. The elemental and mineral oxide composition of the selected CWFs were analysed with x-ray fluorescence method. Furthermore, the raw unfiltered water (from three different common sources), and the filtrates obtained with the CWFs were analysed for their physicochemical, metal ion removability, and microbial correction. The x-ray fluorescence analysis indicated that Al_2_O_3_ and SiO_2_ were the major mineral oxide compositions of the selected CWFs. These metal oxides were present in varying concentrations. The CWFs showed turbidity reduction of 74.28–99.40%, Mn and Fe reduction of 54.04–98.48% and 48.82–97.50% respectively. In addition, the total coliform reduction by the selected CWFs ranged from 2.31 to 76.97%. It was therefore observed from the results that, the efficiency of commercially available CWFs varied in the order BF + AC > CF > PFcs. BF + AC was the most efficient in both physicochemical and microbial correction of all water sources. CWF selection guide for consumers based on different sources of water which considered the physicochemical parameters, biological parameters and Water Quality Index was discussed. This has an implication for regulation and standardization of CWFs.

## 1 Introduction

The use of ceramic water filters (CWFs) has been accepted globally over the past three decades, mostly by developing countries [1,2]. It has been reported that as at the year 2022, 77% of Americans, filter their water at home [3]. These observations are seen in the water purifier market size estimated to be 5.85 billion USD in 2021, and it is expected to be 6.12 billion USD in 2022, and projected to 9.10 billion USD by 2029 [4]. In addition, there are over 50 factories worldwide involved in the production of different designs of CWFs [5]. These products are commercially available in the open markets and retail shops for prospective customers. It has been reported that, consumers have adopted these point-of-use (POU) water treatment technologies due to portable water accessibility issues [6], and it has gained widespread use around the world as indicated earlier [2,7].

The basic raw material for the production of CWFs is clay but other burnout materials such as sawdust, green-fibre (recycled paper fibre), bone char or rice husk are added during production. Over the years, the use of modified CWFs (CWF produced with change in shape or with the addition of activated carbon, processed combustible material or colloidal silver to improve its functionality) have been promoted and in some cases employed as a relief materials in disaster management by NGOs such as Potters for Peace, Pure Home Water, OXFAM, etc., [[8], [9], [10]]. As a result of the filters’ ability to give flow rates greater than 1 l/h [11], reduce turbidity of source water to values > 97.3% [12], reduce microbe after treatment to values > 90% [11,13], reduce waterborne virus after treatment >99.99% [13,14] and reduce the colour of source water to values > 60% [15]. Studies have also indicated that, CWFs produced with activated carbon, and other materials during manufacture, have the ability to effectively reduce the concentration of heavy metal ions such as zinc, nickel, manganese, lead, chromium, arsenite, arsenate and copper [16,17].

In spite of the observed capabilities of some CWFs, other studies have questioned the effectiveness of the POU CWF devices as drinking water treatment technology. Some researchers have doubted [18] the viral and chemical reduction ability of the CWFs. Others [19] have also questioned the long-term efficiency of the CWFs under field conditions and suggested that human factors are crucial for CWF water treatment. Therefore, CWFs require regular maintenance and adequate cleaning as pre-conditions for microbial effectiveness. In addition, a study conducted by Materne and Grüner [20] revealed that, that some of the CWFs studied did not produce water that complied with international standards for drinking water [21], this implies that, not every CWF product improves drinking water quality. Furthermore, there is significant information on the heavy metal leaching capacity of the CWFs in other jurisdictions [[22], [23], [24]]. More specifically, however, there is a dearth of knowledge about the efficiency and particularly, metal removability of commercially available CWFs in the Ghanaian market [25].

These issues aside, it has been observed by the authors that, while many countries worldwide have regulatory standards for bottled and filtered water, there are no regulatory standards for CWFs [25]. In spite the variation in the efficiencies of CWFs and the absence of regulatory guide, their usage has increased, and this makes it imperative to characterize the filters and assess their efficiency.

In view of these, the current study sought to study the efficiency of the commonly used commercially available types of CWFs in Ghana and its neighbouring countries with respect to the common drinking water sources, and generate data sets to guide consumers in the selection of CWFs.

## 2 Materials and methods

### 2.1 Description of sampling location for water

**Fig. 1:**
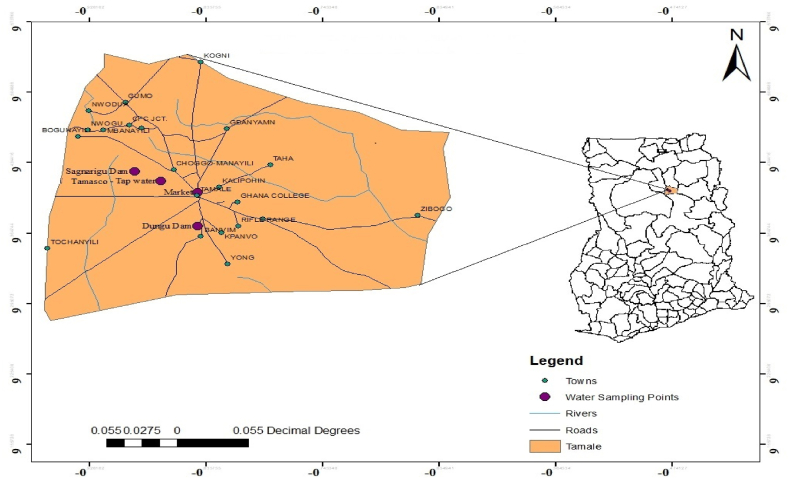
Map showing study area with water sampling locations.

The water sampling area is located within the Tamale metropolis of the Northern Region of Ghana (Fig. 1) with climate temperate of 27.9 °C and annual rainfall of 1111 mm/43.7 inch. Tamale metropolis is considered the fasted growing city in west Africa as at the year 2013, with access to potable drinking water coverage of 50% [26]. It is the third largest city in Ghana but available data suggest that, access to potable water for drinking and other domestic purposes is a challenge.

#### 2.1.1 Water sampling

The water samples were collected from three sources within Tamale. The sampling points were selected based on accessibility, patronage (domestic, agricultural and commercial activities), characteristics, composition and level of pollution. The selected locations were Dungu dam, Sagnarigu Kukoo dam and municipal tap water. The surface water samples, for the purpose of the study referred to as ‘less turbid water’ (LTW) and ‘high turbid water’ (HTW) from Sagnarigu Kukuo and Dungu dams respectively. The surface water samples (Dungu and Sagnarigu Kukuo dams) for POU treatment and water quality analysis (microbiological and physicochemical) were collected mid-stream at depths 20–30 cm directly into sterile 25 L PET gallons [27]. Municipal tap water was sampled from standpipe at Tamale Senior High School campus using standard methods described by Lien et al. [28] with slight modification. The water samples were conveyed to the laboratory and then allowed to settle for a day before filtering with the selected CWFs, as is done locally.

### 2.2 Ceramic water filters (CWFs) sampling

**Fig. 2:**
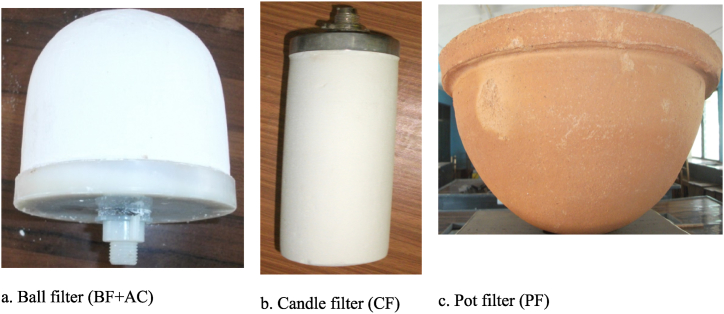
Samples of CWF used for the study.

A survey conducted revealed that there were three types of CWFs available in the Ghanaian market. A classification of the CWFs was done according to the shape, size, and the composition of the filter sets. The common materials used to fabricate the selected filters were clay and combustible materials (rice husk or sawdust). However, the pot filter (PF) was coated with colloidal silver with the following dimensions: 18.8 ± 4.6 cm height, 34.7 ± 4.3 cm inner diameter and 39.5 ± 3.1 cm outer diameter, while the ball filter had a hollow space filled with activated carbon (AC) with the following dimensions: 8.2 ± 2.4 cm height, 8.9 ± 3.3 cm inner diameter and 10.5 ± 2.4 cm outer diameter. In addition, the candle filter had a hollow space in the middle with the following dimensions: 8.0 ± 2.5 cm height, 5.3 ± 2.0 cm inner diameter and 5.6 ± 1.3 cm outer diameter. The selected filters for the study were, ball filter (BF + AC) as shown in Fig. 2a, hollow candle filter (CF) as shown in Fig. 2b and pot filter (PFcs) as shown in Fig. 2c.

### 2.3 Porosity analysis of CWFs

Porosity of the commercially available CWFs were determined using the water absorption test (direct) method, and the determination was repeated five times for each CWF. The destructive method was used where three samples of each CWF (BF + AC, CF and PFcs) weighing (263 ± 0.58 g, 315.33 ± 0.88 g and 4000 ± 28.87 g) were taken and the average porosity of the samples were estimated as the apparent porosity of filters. The samples were weighed when dry in air (M_1_) then saturated in distilled water at room temperature for 20 h. The water with the samples was then boiled for about 2 h (for PFcs and CF) while boiled water was added to BF + AC. The water was allowed to cool to room temperature for another 20 h. This was done to ensure that the air in the open pores of the filter samples was replaced by the distilled water. The soaked samples were weighed under distilled water (M_2_) then removed, and the surface wiped with tissue paper followed by weighing in air (M_3_). The weight of the wire was subtracted from the value obtained while determining the weight of the sample suspended in water. Apparent porosity (P) was then calculated using the expression stated in equation (1.0) below [29].

1.0 $$P=100\times \left(\frac{M_{3}-M_{1}}{M_{3}-M_{2}}\right)$$

where; M_3_ is the weight of the specimen when saturated in water; M_1_ is the weight of the dry specimen; M_2_ is the weight of the sample underwater.

### 2.4 Filtration experiment

**Fig. 3:**
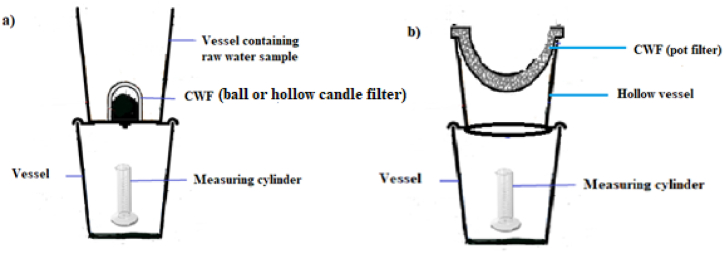
Schematic diagram of setup for filtration experiments: Setup for (a) ball or hollow candle filter and (b) pot filter.

Filtration experiments were conducted for the three commercially available CWFs. Three set of each filter (ball, candle and pot) as shown in Fig. 2 (a, b & c respectively) were soaked in distilled water overnight. The CWFs were thoroughly washed with distilled water which was in line with the guidelines of manufacturers before use. This is done to avoid possible leachates. The setup of the filtration experiment was designed to have filter (pot) raised above the collection vessel to prevent the obstruction of fluid flow out of the sides of the filter (Fig. 3b). The pot filter was placed in a hollow bowl with an opening underneath. The hollow bowl containing the pot filter was placed on a vessel (receptacle) with a measuring cylinder for collection of the filtrate (water samples).

The ball and candle filters were placed in a vessel and screwed into a hole at the centre of the vessel (Fig. 3a). The vessels containing the filter elements were placed on a vessel (receptacle) with a measuring cylinder for collection of the filtrate (water samples). For the nine setups, water samples (TW, LTW and HTW) were poured into the container with the filter and the water was allowed to percolate through the pore spaces of the filters into the measuring cylinder in the receptacle. The volume of filtrates were read at thirty (30) minutes interval with the corresponding height in the measuring cylinder as indicated in an experiment conducted by Ref. [30]. The procedure was repeated three times, separately, for all CWF types.

The flow rate was calculated using the relationship described in equation (2.0) from the volume of filtered water and the time of filtration.

2.0 $$Flow\;rate=\frac{Volume\;of\;water\;measured\;at\;time\;t\;\left(mL\right)}{Elapsed\;time\;\left(t\right)from\;start\;of\;test\;\left(Hours\right)}$$

### 2.5 Physicochemical and microbiological analysis

The water samples (raw water and filtrates obtained using the CWFs) for analysis were collected with sterilized PET bottles of 500 ml capacity, and the determination of physicochemical parameters were conducted using standard methods for the examination of water and wastewater described by AHPA [31] with slight modifications. The pH, EC, temperature and TDS of raw water samples and filtrates were measured directly by the use of Pancellent (TES-PH-ATDS) TDS, EC and Temperature meter in-situ, and in the laboratory. The turbidity of the water samples was measured by the use of HACH 2100 AN turbidimeter in the laboratory. Also, the total hardness of the samples was determined by the titrimetric method. Aliquot of the water samples were titrated against 0.01 M EDTA solution with replications. The average titre values were recorded and used in the calculation of the total (Ca and Mg) hardness of the samples. The divalent metal ions (Fe and Mn) were determined from the water sample by the UV–Vis spectrometric method.

Water samples for microbiological analysis were collected in sterilized non-reactive PET bottles of 500 ml capacity. The samples were collected before and after filtration for all water sample types. The total coliform count was performed using the pour plate method. 1 ml of each sample was kept in a Petri dish containing MarConey Agar, rotated for uniform mixing and allowed in an incubator for at 37 °C for 24 h, after which the colonies were counted and recorded [31].

### 2.6 Determination of elemental and metal oxide compositions of CWFs

The elements and metal oxides composition of the commercially available CWFs were assessed. The CWFs were separated into their components and pulverised with a clean mortar and pestle. The pulverised samples were analysed for their elemental and metal oxide composition with X-ray fluorescence spectrometer (Vanta VMR).

### 2.7 Water quality index (WQI) computation

The water quality index (WQI) method was adopted to rank the efficiency of the commercially available CWFs in the country. The study used the seven physicochemical characteristics (turbidity, EC, TDS, Fe, Mn, total hardness and pH) as indicated in Table 2, Table 3 for the computation of the WQI scores for raw water samples and filtrates of CWFs. Weighted arithmetic index method [32,33] were adopted for the computation of the WQI. Water quality index score was computed using equations 3, 4 as follows;

3 $$q_{n}=100\left[V_{n}-V_{o}\right]/\left[S_{n}-V_{o}\right]$$

Where $q_{n}$ = quality rating for nth water quality parameter $V_{n}$ = estimated value of the nth water quality parameter in pure water $S_{n}$ = standard permissible value of the nth water quality parameters.

4 $$W_{n}=\frac{k}{S_{n}}$$

Where k = constant of proportionality W_n_ = unit weight for the nth parameter WQI values of the raw water and filtrates of the selected CWFs were calculated using the physicochemical parameters with the aid of the overall formula of equation (5.0):

5.0 $$WQI=\frac{{\sum W}_{n}q_{n}}{{\sum W}_{n}}$$

### 2.8 Statistical analysis

Data analyses were performed with the IBM statistical package for the social sciences (IBM SPSS version 23). The physicochemical data generated from water samples and filtrates of CWFs were subjected to analysis of variance (ANOVA) using the general linear model procedure. Least significant difference (LSD) test was used to determine the differences among the filtrates obtained using the CWFs and the raw water samples based on P = 0.05.

## 3 Result and discussion

### 3.1 Chemical composition of CWFs

**Fig. 4:**
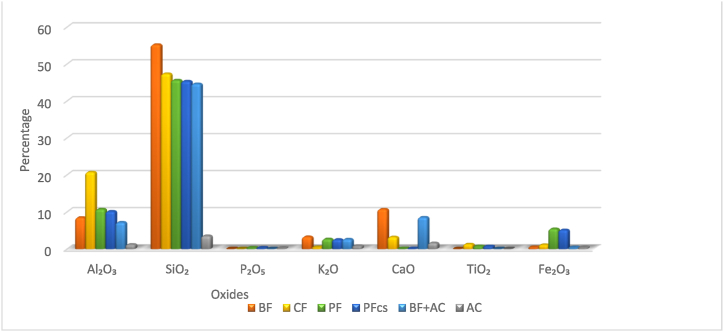
Composition of oxides in selected CWFs.

The major oxides of elements in all the CWFs were silica (SiO_2_), and alumina (Al_2_O_3_) followed by Fe_2_O_3_, K_2_O and CaO which were present in moderate concentrations. The rest of the oxides were present in small quantities. The concentration of CaO is well known [34] to decrease the red colour iron in clay material and this may explain the observed white to the off white colour of BF + AC and CF (CWFs). The quantity of alumina and silica are 10.14% and 45.34% in PFcs, 7.18% and 44.62% in BF + AC and 20.73% and 47.34% in CF (Fig. 4). The red colour of PFcs could be attributed to the use of iron-rich clay for its fabrication as also observed other researchers [24].

The major elements common to all the CWFs were silicon, aluminium, iron and calcium (Table 1). Trace quantities of manganese, and lead were equally present and titanium was present only in PFcs (4419 mg/kg) and CF (7741 mg/kg). Arsenic and molybdenum were either undetectable or present in negligible quantities. All the elements apart from silicon, aluminium and calcium could be injurious to consumers if they leach into filtrates, and consumed over time. Therefore, the continuous monitoring of the levels of elemental composition of raw materials of CWFs is to be considered an essential quality control parameter. The trace concentrations of silver found in PFcs could be due to the silver coating on the filter materials after production since it was absent in the pot filter without colloidal silver coating (PF) [24].

**Table 1:** Concentration of elements in CWFs.

CWF	Element (mg/kg) ± STD							
	Fe	Ca	Ti	Mn	Pb	As	Mo	Ag
PF	3.8 × 10^4^ ± 1 × 10^3^	449 ± 9	4.5 × 10^3^ ± 87	270 ± 10	11 ± 0.26	9 ± 0.34	bdl	bdl
PFcs	3.6 × 10^4^ ± 1 × 10^3^	540 ± 10	4.4 × 10^3^ ± 100	237 ± 10	11 ± 0.26	8 ± 0.44	bdl	215 ± 10
BF	3.4 × 10^3^ ± 200	7.6 × 10^4^ ± 1 × 10^3^	bdl	156 ± 20	47 ± 0.61	bdl	bdl	Bdl
BF + AC	2.7 × 10^3^ ± 100	6.1 × 10^4^ ± 1 × 10^3^	Bdl	118 ± 10	34 ± 1	bdl	bdl	67 ± 0.1
CF	7.9 × 10^3^ ± 100	2.3 × 10^4^ ± 1 × 10^3^	77 × 10^3^ ± 200	66 ± 1	26 ± 3	4 ± 0.17	6 ± 0.3	bdl

**NB:** bdl = below detectable limit.

Apparent porosity of commercially available CWFs.

The porosity of the three commercially available CWFs were 58.55%, 60.61% and 75.00% for the BF + AC, CF and PFcs respectively. The apparent porosity of the CWFs were in the order, BF + AC < CF < PFcs. Which indicated that PFcs was of the highest porosity and BF + AC was the least. The result of the porosity were in line with other studies [35,36]. The variations in the apparent porosity of the CWFs could be attributed to the quantity of burnout material added to the clay during production of the filter, that is the variations in porosity is directly proportional to the quantity of combustible matter added [36,37]. However, the apparent porosity of the CWFs were inversely proportional to the filters ability to remove total coliform and suspended particles. It is understood by the authors that, the silver nitrate coatings on the pot filter (PFcs) compensate for the relatively high porosity of the material. Since low porosity is good for the removal of microbes, one would expect these relationships at all times. In this study it was noted that the porosity as well as the materials used in the production of the CWF can influence the amount of coliform it can remove from a given water. In this study, coliform count of the water samples was in the order, HTW > LTW > TW (Table 2). However, the relative efficiency of the CWFs in the removal of did not occur in order of porosity of the CWFs, except in the case of the HTW as shown below.

**TW:***CF* > *PFcs* > *BF* + *AC*; **LTW:** *PFcs* > *BF* + *AC* > *CF*; **HTW:** *BF* + *AC* > *CF* > *PFcs*.

The observation indicates that the modification of CWFs have effect on their efficiency in the effective treatment of water. In this case the PFcs was coated with silver, the BF-AC had the least porosity, so the authors are of the view that the flow rate and coliform count of the raw water are factors which account for the deviation from the theorised inversed relationship between porosity and coliform removal efficiency.

#### 3.1.1 Characteristics of raw water

The characteristics of the raw water treated with the CWFs are presented in Table 2. Apart from the presence of total coliform for tap water (TW), the physicochemical characteristics of the water samples were all within the acceptable limits recommended by WHO [21]. On the other hand, the concentration of turbidity, iron, manganese and total coliform of the less turbid water (LTW) and high turbid water (HTW) samples were higher than the maximum acceptable limits recommended by WHO [21]. These results provide the justification for treating these water samples to make them fit for consumption without any adverse effects.

**Table 2:** Physicochemical characteristics of raw water.

Parameter	Sample			WHO standard
	TW	LTW	HTW	
Turbidity (NTU)	1.49 ± 0.1	637 ± 8.72	1800 ± 26.46	5
E.C. (μS/cm)	156.1 ± 6.1	185.7 ± 3.58	179.6 ± 3.61	1000
TDS (mg/l)	78.0 ± 4.1	92.8 ± 4.35	89.8 ± 4.55	1000
Fe (mg/l)	0.06 ± 0.001	1.74 ± 0.08	5.08 ± 0.04	0.3
Mn (mg/l)	bdl	0.175 ± 0.02	1.982 ± 0.05	0.4
Total Hardness (mg/l) pH	42 ± 0.36	31 ± 1.99	16 ± 2.08	300
pH	6.80 ± 0.75	7.13 ± 0.65	6.66 ± 0.55	6.5–8.5
T. Col. (cfu/ml)	5 ± 1	115 ± 1	178 ± 1	0

NB: bdl = below detectable limit.

### 3.2 Characteristics of filtered water

The results of the physicochemical characteristics of filtrates (filtered water) with the selected CWFs are as presented in Table 3. Comparatively, the pH of the raw water samples, and filtrates ranged from 6.66 to 7.13 and 7.23 to 7.65, respectively. The pH of all the filtrates were generally higher than those of the raw water samples, irrespective of the CWF used for filtration. This corroborates the observations of other researchers [24,38].

**Table 3:** Physicochemical characteristics of Filtered water.

Water samples	CWFs	Physicochemical Parameters				
		pH	E.C. (μS/cm)	TDS (mg/l)	Total Hardness (mg/l)	Turbidity (NTU)
**TW**	BF + AC	7.57 ± 0.30	119.00 ± 6.66	82.00 ± 2.73	32.00 ± 2.30	1.55 ± 0.78
	CF	7.48 ± 0.17	218.00 ± 5.30	147.00 ± 3.46	42.00 ± 1.90	7.60 ± 0.80
	PFcs	7.23 ± 0.04	140.00 ± 6.25	57.00 ± 3.46	28.00 ± 2.65	5.80 ± 0.77
**LTW**	BF + AC	7.28 ± 0.03	469.00 ± 4.58	234.20 ± 3.85	50.00 ± 3.61	5.47 ± 1.62
	CF	7.25 ± 0.03	450.00 ± 5.65	225.00 ± 3.00	45.00 ± 3.46	11.50 ± 3.70
	PFcs	7.23 ± 0.03	456.00 ± 5.57	228.00 ± 5.69	35.00 ± 2.30	19.50 ± 4.80
**HTW**	BF + AC	7.65 ± 0.03	421.00 ± 4.70	210.50 ± 2.17	36.00 ± 3.00	10.80 ± 1.86
	CF	7.54 ± 0.08	437.00 ± 5.61	218.50 ± 2.17	27.00 ± 3.00	387.00 ± 10.43
	PFcs	7.36 ± 0.08	192.00 ± 4.35	96.00 ± 3.73	29.00 ± 2.73	463.00 ± 13.11
**WHO standards**		6.5–8.5	1000	1000	300	5

The elevation of pH of the filtrates could be attributed to the adsorption of charged species which is a function of the surface charge of clay (raw material of CWFs) [39]. The pH of the filtrates was within the recommended range of 6.5–8.5 [40]. Ranking CWFs according to their efficiency give the order, BF + AC > CF > PFcs.

The electrical conductivity (E.C) of the samples of raw water and their filtrates ranged from 156.1 to 185.7 and 140.0 to 469.0 μS/cm; respectively. The E.C. of all the treated water samples (except two), were approximately higher than that of the raw water irrespective of the CWF used for the filtration. The E.C. of the filtrate from PFcs, CF and BF + AC ranged from 140.0 to 456.0, 218.0 to 450.0 and 119.0 to 469.0 μS/cm respectively. The increase in E.C values of filtrates with CWFs compared to raw water corroborates similar observation by Kar and Gupta [41]. However, E.C values of the filtrates were lower than the maximum permissible value of 1000 μS/cm [21]. Comparing CWFs efficiency of E.C based on the results give, PFcs > BF + AC > CF in terms of correction of filtrate.

The TDS of the raw and filtrates ranged from 78.0 to 92.8 and 57.0–228.0 mg/l, respectively. The TDS of all the treated water samples but one (57.00 ± 3.46), were higher than that of the raw water irrespective of the CWF used for the filtration. This agrees with other research observations [41]. The TDS of the filtrates were much lower than the maximum recommended 1000 mg/l [21]. However, the reduction by the filters is in the order, PFcs > BF > CF.

The total hardness of raw and filtrates water ranged from 16 to 42 and 27–50 mg/l, respectively. The values of the filtrates were higher than that of the raw water samples except for TW. The observed elevation of the total hardness of the filtrates with CWF could be as a result of leached ions (Mg^2+^ and/or Ca^2+^) into the filtrates [24]. In addition, the reduction in total hardness values of TW may be due to adsorption of either calcium and/or magnesium ions by the filter [42]. The highest total hardness value obtained for the filtrates was 50 mg/l which is very low compared to the maximum permissible value of 300 mg/l [21]. The observations made for total hardness values of treated water samples corroborates with similar observation made by other researchers [41]. However, comparing the efficiency of the CWFs for total hardness correction of a drinking water, in the order, BF + AC > CF > PFcs.

The turbidity of TW samples treated with the selected CWFs ranged from 1.55 to 7.60 NTU, which suggests an increase in turbidity values compared to the turbidity of the raw TW sample (Table 3). The increment in the turbidity value could be attributed to leaching of colloidal solids present in raw water or fine particles of the filter materials. This could be as a result of the first-time usage of CWFs as indicated in study by Ref. [24]. The turbidity of the filtrates from PFcs, CF and BF + AC ranged from 19.5 to 463, 11.5 to 387 and 5.47 to 10.8 NTU; respectively. The reduction in turbidity observed for LTW and HTW could be as a result of suspended solids of the raw surface water samples removed through physical processes such as clogging, inertia and adsorption [42]. Turbidity of filtrates of CWFs were lower as compared to the raw water samples. This could be due to clogging of pore spaces, which showed in reduction of flow rate of the filters. In addition, turbidity removability for LTW was in the order of BF + AC > CF > PFcs with percentage removal of 99.14, 98.19 and 96.94 respectively. Furthermore, turbidity removability for HTW was in the order of BF + AC > CF > PFcs with percentage removal of 99.40, 78.50 and 74.28 respectively. The percentage turbidity reduction of the selected CWFs were in line with other studies [15,24,43]. From the analysis of turbidity reduction by CWFs for LTW and HTW, it could be concluded that the order is BF + AC > CF > PFcs. Therefore, BF + AC is the most efficient in turbidity removal from surface water as compared to other selected CWFs. The selected CWFs showed physicochemical corrections to some varying extent, the observed correctional efficiency of the filters was noted to be in the order BF + AC > CF > PFcs.

### 3.3 Statistical evaluation of CWFs

The hypothesis was analysed using one-way analysis of variance in comparing the physicochemical characteristics of the raw water sample (TW, LTW and HTW) and filtrates of the commercially available CWFs (BF + AC, CF and PFcs) at 0.05 level of significance. The results are presented in Table 4, Table 5, Table 6, Table 7, Table 8.

**Table 4:** ANOVA of the pH of the raw water samples and the filtrates of CWFs.

		Sum of Squares	df	Mean Square	F	Sig.
pH of TW	Between Groups	1.070	3	.357	1426.400	.000
	Within Groups	.002	8	.000		
	Total	1.072	11			
pH of LTW	Between Groups	.038	3	.013	31.688	.000
	Within Groups	.003	8	.000		
	Total	.041	11			
pH of HTW	Between Groups	1.780	3	.593	3390.143	.000
	Within Groups	.001	8	.000		
	Total	1.781	11

**Table 5:** ANOVA of the E.C of the raw water samples and the filtrates of CWFs.

		Sum of Squares	df	Mean Square	F	Sig.
EC of TW	Between Groups	11660.923	3	3886.974	7.309	.011
	Within Groups	4254.707	8	531.838		
	Total	15915.629	11			
EC of LTW	Between Groups	167806.103	3	55935.368	74332.714	.000
	Within Groups	6.020	8	.753		
	Total	167812.123	11			
EC of HTW	Between Groups	178053.360	3	59351.120	26261.558	.000
	Within Groups	18.080	8	2.260		
	Total	178071.440	11

**Table 6:** ANOVA of the TDS of the raw water samples and the filtrates of CWFs.

		Sum of Squares	df	Mean Square	F	Sig.
TDS of TW	Between Groups	13626.000	3	4542.000	1816.800	.000
	Within Groups	20.000	8	2.500		
	Total	13646.000	11			
TDS of LTW	Between Groups	41911.440	3	13970.480	6967.820	.000
	Within Groups	16.040	8	2.005		
	Total	41927.480	11			
TDS of HTW	Between Groups	44513.340	3	14837.780	57622.447	.000
	Within Groups	2.060	8	.258		
	Total	44515.400	11

**Table 7:** ANOVA of the total hardness of the raw water samples and the filtrates of CWFs.

		Sum of Squares	df	Mean Square	F	Sig.
TH of TW	Between Groups	456.000	3	152.000	86.857	.000
	Within Groups	14.000	8	1.750		
	Total	470.000	11			
TH of LTW	Between Groups	723.000	3	241.000	241.000	.000
	Within Groups	8.000	8	1.000		
	Total	731.000	11			
TH of HTW	Between Groups	618.000	3	206.000	82.400	.000
	Within Groups	20.000	8	2.500		
	Total	638.000	11

**Table 8:** ANOVA of the turbidity of the raw water samples and the filtrates of CWFs.

		Sum of Squares	df	Mean Square	F	Sig.
Turbidity (TW)	Between Groups	85.363	3	28.454	11050.175	.000
	Within Groups	.021	8	.003		
	Total	85.383	11			
Turbidity (LTW)	Between Groups	878762.882	3	292920.961	292877.029	.000
	Within Groups	8.001	8	1.000		
	Total	878770.883	11			
Turbidity (HTW)	Between Groups	5502871.440	3	1834290.480	222337.566	.000
	Within Groups	66.000	8	8.250		
	Total	5502937.440	11			

The result further revealed that the calculated F-ratio obtained for the physicochemical characteristics (pH, E.C, TDS, total hardness and turbidity) for TW is 1426.40, 7.31, 1816.80, 86.86 and 11050.18 respectively with p-value <0.001 (except EC which is 0.011) at 0.5 level of significance with 3 and 8° of freedom. The analysis indicates that, the F-values for all filtrate of CWFs for TW are significant with exception of E.C. This suggests that the null hypothesis which states that CWFs are not effective for the correction of the physicochemical characteristics of TW is rejected. Since CWFs are effective in the treatment of the physicochemical characteristics of TW, the sources of the difference were determined using Fisher Least Significant Difference (LSD) Post Hoc Test multiple comparison analysis. The result of the LSD revealed that the mean differences for change in pH of the filtrates of the CWFs compared to the raw unfiltered TW is −0.77, −0.68, and −0.43 for BF + AC, CF and PFcs indicating an elevation in the pH as observed in Table S3.0. Also, the LSD showed that the mean differences for change in E.C of filtrates of the CWFs compared to the TW is 10.43 (not sig where p-value >0.001), −61.90 (sig where p-value <0.001) and 16.00 (not sig where p-value >0.005) for BF + AC, CF and PFcs suggesting changes that occurred in BF + AC and PFcs could not affect the E.C of the filtrates yet change in EC of CF was significant enough. In addition, the LSD indicates that the mean differences for change in TDS of filtrates of the CWFs compared to the TW is −4.00 (not sig where p-value >0.005), −69.90 (sig where p-value <0.001) and 21.00 (sig where p-value <0.001) for BF + AC, CF and PFcs indicating a significant change in the TDS of filtrates of CF and PFcs Whilst the filtrates of BF + AC showed no significant changes in TDS. Also, the LSD showed that the mean differences for change in total hardness of filtrates of the CWFs compared to the TW are 10.00 (sig where p-value <0.001), 0.00 (not sig where p-value >0.005) and 14.00 (sig where p-value <0.001) for BF + AC, CF and PFcs. This suggests that two of the CWFs (i.e., BF + AC and PFcs) significantly affected the total hardness of the filtrate, yet the filtrates of CF showed no significant changes in total hardness. Finally, the LSD showed that the mean differences for change in turbidity of filtrates of the CWFs compared to the TW are −0.60 (sig where p-value >0.005), −6.11 (sig where p-value <0.001) and −4.31 (sig where p-value <0.001) for BF + AC, CF and PFcs. This suggests that two of the CWFs (i.e., CF and PFcs) significantly affected the turbidity of the filtrate by slightly increasing as observed in Table 3, yet the filtrates of BF + AC showed no significant changes in turbidity correction of the filtrate.

The ANOVA result of LTW also revealed that the calculated F-ratio obtained for the physicochemical characteristics (pH, E.C, TDS, total hardness and turbidity) is 31.69, 74332.71, 6967.82, 241.00, and 292877.029 with p-value <0.001 at 0.5 level of significance with 3 and 8° of freedom (Table 4, Table 5, Table 6, Table 7, Table 8). The analysis suggested that F-values for all filtrate of CWFs for LTW are significant. This also indicates that the null hypothesis which states that CWFs are not effective for the correction of the physicochemical characteristics of LTW is rejected. Since CWFs are effective in the treatment of the physicochemical characteristics of LTW, the sources of the difference were determined using Fisher Least Significant Difference (LSD) Post Hoc Test multiple comparison analysis. The result of the LSD revealed that the mean differences for change in pH of the filtrates of the CWFs compared to the raw unfiltered LTW is −0.15, −0.12, and −0.10 for BF + AC, CF and PFcs indicating an elevation in the pH as observed in Table S3.0. The observed mean differences indicate significant difference with p-value <0.001. Also, the LSD showed that the mean differences for change in E.C of filtrates of the CWFs compared to the raw LTW is −283.30, −264.30 and −270.30 for BF + AC, CF and PFcs and significant with p-value <0.001. The changes that occurred confirms the elevation of the E.C values of filtrate as indicated Table S3.0. In addition, the LSD indicates that the mean differences for change in TDS of filtrates of the CWFs compared to the LTW is −141.40, −132.20 and −135.20 for BF + AC, CF and PFcs. The changes that occurred confirms the elevation of the TDS values of filtrate of CWFs as indicated Table S3.0. However, there was significant differences in the changes observed in TDS with p-value <0.001. Furthermore, the LSD showed that the mean differences for change in total hardness of filtrates of the CWFs compared to the LTW are −19.00, −15.00 and −4.00 for BF + AC, CF and PFcs. The changes that occurred with a significant difference in the changes observed in total hardness with p-value <0.001 confirms the elevation of the total hardness values of filtrate of CWFs as indicated Table S3.0. Finally, the LSD showed that the mean differences for change in turbidity of filtrates of the CWFs compared to the LTW are 631.53, 625.50 and 617.50 for BF + AC, CF and PFcs. The changes that occurred confirms the total reduction of the turbidity values of filtrate of CWFs as observed in Table S3.0. There changes observed in turbidity was significant.

Finally, the result obtained for CWFs effectiveness in the treatment of HTW revealed that the calculated F-ratio obtained for the physicochemical characteristics (pH, E.C, TDS, total hardness and turbidity) is 3390.14, 26261.56, 57622.45, 82.40, and 222337.57 with p-value <0.001 at 0.5 level of significance with 3 and 8° of freedom (Table 4, Table 5, Table 6, Table 7, Table 8). The analysis suggested that F-values for all filtrate of CWFs for HTW are significant. This also indicates that the null hypothesis which states that CWFs are not effective for the correction of the physicochemical characteristics of HTW is rejected. Since CWFs are effective in the treatment of the physicochemical characteristics of HTW, The sources of the difference were determined using Fisher Least Significant Difference (LSD) Post Hoc Test multiple comparison analysis. The result of the LSD revealed that the mean differences for change in pH of the filtrates of the CWFs compared to the raw unfiltered HTW is −0.99, −0.88, and −0.70 for BF + AC, CF and PFcs which indicates slight elevation in the pH as observed in Table S3.0. Is significant difference with p-value ≤0.001. Moreover, the LSD showed that the mean differences for change in E.C of filtrates of the CWFs compared to the HTW is −241.40, −257.40 and −12.40 for BF + AC, CF and PFcs is significant (p-value <0.001). This confirms the observed changes in E.C values of filtrate as indicated Table S3.0. In addition, the LSD indicates that the mean differences for change in TDS of filtrates of the CWFs compared to the HTW is −120.70, −128.70 and −6.20 for BF + AC, CF and PFcs. The changes that occurred confirms the elevation of the TDS values of filtrate of CWFs as indicated Table S3.0. There were significant differences in the changes observed in TDS with p-value <0.001. Furthermore, the LSD showed that the mean differences for change in total hardness of filtrates of the CWFs compared to the HTW are −20.00, −11.00 and −13.00 for BF + AC, CF and PFcs. The implication is that the observed increase in the total hardness values of filtrate of CWFs is significant with p-value ≤0.001. Finally, the LSD showed that the mean differences for change in turbidity of filtrates of the CWFs compared to the HTW are 1789.20, 1413.00 and 1337.00 for BF + AC, CF and PFcs, with a p-value >0.001, indicating and confirming the observed reduction in turbidity From the ANOVA results of the three water samples against the CWFs, it was observed that, commercially available CWFs were efficient in the treatment of the water samples with slight variation which could be as a result of the intrinsic properties, porosity, and surface area of the CWFs. The statistical evaluation of the physicochemical characteristics of the filtrate with CWFs (BF + AC, CF and PFcs) for the TW, LTW and HTW showed significance difference which implies that the filters were effective in the treatment of water irrespective of the level of contamination in source water. Also, the ANOVA revealed that turbidity removability of commercially available CWFs increases with increase of the total suspended solids in the source water (HTW > LTW > TW).

#### 3.3.1 Metal removability of CWFs

**Fig. 5:**
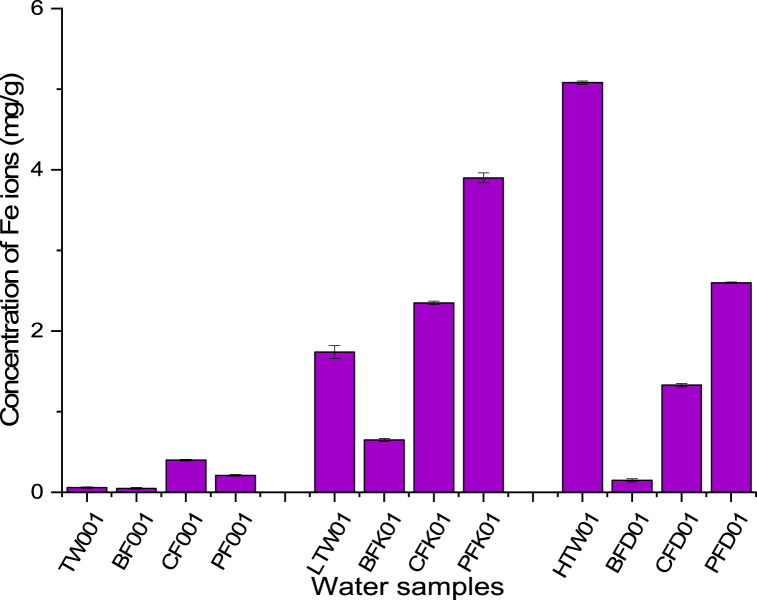
Concentration of Fe^2+^ of raw water sample and filtrates of selected CWFs.

**Fig. 6:**
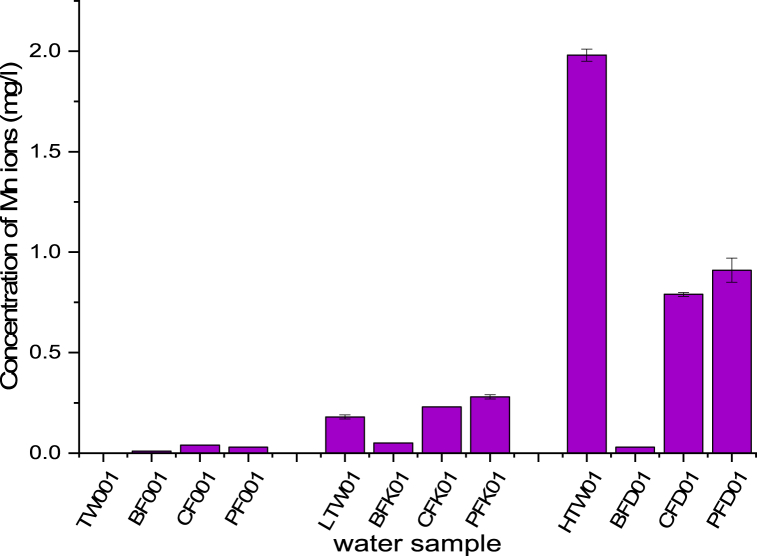
Concentration of Mn^2+^ of raw water sample and filtrates of selected CWFs.

Comparing concentration of metal ions in raw and filtered water obtained with the CWFs for HTW gave 5.08 mg/l (Fe) and 1.98 mg/l (Mn). Metal ion concentration for Mn (0.03 mg/l with BF + AC, 0.79 mg/l with CF and 0.91 mg/l with PFcs) and Fe (0.15 mg/l for BF + AC, 1.33 mg/l for CF and 2.60 mg/l for PFcs) varied with the filter type (Fig. 5). Although the concentration of metal ions of the HTW filtered with CWFs were above the maximum permissible value set for drinking water by Ref. [21], the CWFs indicated percentage removability in the range of 54.04–98.48% for Mn and 48.82–97.5% for Fe (Fig. 5, Fig. 6) which was in line with other studies [24,42,43]. The efficiency of metal removal was in the order of BF + AC > CF > PFcs. BF + AC gave the highest removability for all metal ions followed by CF with PFcs as the least. Metal ions in solution could be removed through adsorption processes, which was evident in the reduction of metal ion concentration of the water filtrates obtained using the CWFs. This reduction may be as a result of the addition of activated carbon (in the case of BF + AC) which could enhance the metal adsorption characteristics of the filter. Metal ion removability of CWFs increased with continuous usage of the filter especially for PFcs and CF, although the filters indicated some extent of leaching ability for a while [24]. However, the metal ions responsible for water hardness (Ca^2+^ and Mg^2+^) as observed by Martins, Pardo, and Boaventura [44] do not affect the adsorption of other metal ions in solution.

**Fig. 7:**
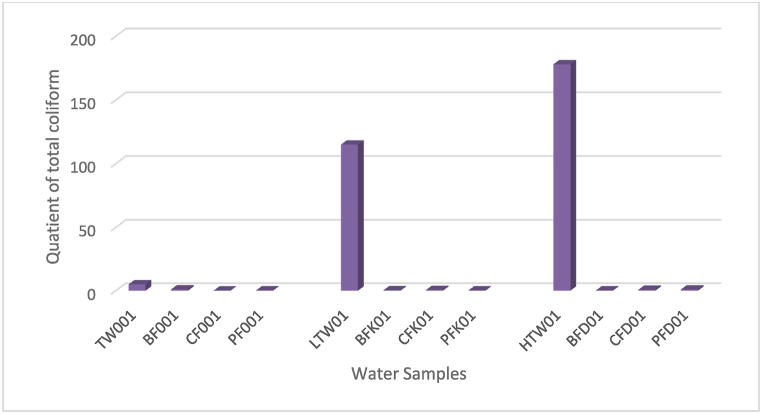
Levels of total coliform in water before and after filtration with CWFs.

The CWFs were able to remove microbes (Fig. 7) in line with the results of other researchers [11,42,43,[45], [46], [47], [48]]. This capability hinges on the composition, pore size and incorporation of antimicrobial compound like colloidal silver (PFcs). The capability of CWFs to remove microbes varied with microbial load in the source water. For the contaminated water samples, CWFs displayed total coliform removal capacity in the order: CF > PFcs > BF + AC and CF > BF + AC > PFcs for LTW and HTW, respectively. The count of total coliform (TC) of raw water and filtrates with CWFs (Fig. 7) ranged from 115 to 178 and 29–173 cfu/ml, respectively. BF + AC indicated total coliform counts for LTW and HTW as 41 and 41 cfu/ml respectively. CF also indicated TC count for LTW and HTW as 64 and 127 cfu/ml respectively. Finally, PFcs indicated TC count for LTW and HTW as 29 and 173 cfu/ml respectively. For BF + AC filter, it was noted that as microbial load in raw water sample increase, the effectiveness to reduce the microbial count also increases. This is observed in percentage of TC removed by BF + AC filter, 63.35% (LTW) and 76.97% (HTW). It could be stated that total coliform removal capacity for BF + AC is in the order HTW > LTW, this suggests that, the filter material could have removed microbes from water based on clogging, inertia and adsorption [42].

The turbidity correction strength of the filter appears to be enhanced from clogging of filter pores in water with high colloidal substances as observed for the HTW. For PFcs and CF, as microbial load in water sample increases, the effectiveness of the CWFs to reduce microbial count decreases. The observed percentage of TC removed from raw water samples by PFcs and CF were LTW (74.78 and 44.35%) and HTW (28.65 and 2.81%), respectively. However, analysis of the XRF data for BF + AC composition indicated the presence of silver (Table 1), which may depend on its form when exposed to water serve as a bactericide. In addition, the colloidal silver coat of CWF (PFcs) may be exhausted due to leaching as observed by other studies [24], and the method of application [5]. Additionally, CWFs that depend solely on metal toxicity (colloidal silver) to remove microbes could have the dead cell of the organisms in the filtrates not necessarily through size exclusion. Comparative analysis of flow rate of CWFs indicated that PFcs had the highest flow rate followed by BF + AC and CF, which is directly proportional to porosity but inversely proportional to microbial removal ability of the filters during the treatment of HTW.

### 3.4 Chemical and microbial data as a guide for selection of CWFs

Ceramic water filters are regarded as one of the most powerful filter media. They have been used for a long time and has gain the long-standing history of effectiveness in water treatment.

Bacteria, total dissolved solids, virus, turbidity, protozoa, cysts, etc., have been known to be capable of being removed from water with the aid ceramic water filters. However, aside the handling related issues such as lack of drop-proof, and inability to remove some chemical contaminants, the most obscured challenge is the absence of chemical-oriented application by users. The limitations spoken of by users are chemical affinity related. This knowledge is what has led to modification of the CWFs to include activated carbon block, rice husk, etc., during their production.

The data obtained in this study was used to prepare a CWF selection guide based on the physicochemical and bacteriological parameters of three major sources of water, using Ghana as a case study (Table 9, Table 10, Table 11). The colours in the table are used to indicate the order of choice based on characteristics of the water type to be filtered.

**Table 9:** CWF selection guide for tap water (TW).

Parameters		CWFs	Percentage Correction	Ratio of Leachate to actual of Raw Water
Physico-chemical	Turbidity (NTU)	BF + AC	0.00	0.004
		PFcs	0.00	2.893
		CF	0.00	4.101
	E.C (μS/cm)	BF + AC	23.77	0.000
		PFcs	10.31	0.000
		CF	0.00	0.396
	Total Hardness (mg/l)	PFcs	33.33	0.000
		BF + AC	23.81	0.000
		CF	0.00	0.000
	TDS (mg/l)	PFcs	26.92	0.000
		BF + AC	0.00	0.513
		CF	0.00	0.885
Chemical	pH	BF + AC	0.00	0.113
		CF	0.00	0.100
		PFcs	0.00	0.632
	Fe^2+^ (mg/l)	BF + AC	16.67	0.167
		PFcs	0.00	2.500
		CF	0.00	5.667
	Mn^2+^ (mg/l)	BF + AC	0.01	0.000
		PFcs	0.03	0.000
		CF	0.04	0.000
Biological	Coliform (cfu/ml)	CF	100.00	0.000
		PFcs	80.00	0.000
		BF + AC	0.00	0.000

**Table 10:** CWF selection guide for less turbid water (LTW).

Parameters		CWFs	Percentage Correction	Ratio of Leachate to actual of Raw Water
Physico-chemical	Turbidity (NTU)	BF + AC	99.14	0.000
		CF	98.19	0.000
		PFcs	96.94	0.000
	E.C (μS/cm)	CF	0.00	1.420
		PFcs	0.00	1.456
		BF + AC	0.00	1.526
	Total Hardness (mg/l)	PFcs	0.00	0.110
		CF	0.00	0.452
		BF + AC	0.00	0.613
	TDS (mg/l)	CF	0.00	1.425
		PFcs	0.00	1.457
		BF + AC	0.00	1.524
Chemical	pH	BF + AC	0.00	0.021
		CF	0.00	0.017
		PFcs	0.00	0.140
	Fe^2+^ (mg/l)	BF + AC	62.64	0.000
		CF	0.00	0.351
		PFcs	0.00	1.241
	Mn^2+^ (mg/l)	BF + AC	71.43	0.000
		CF	0.00	0.314
		PFcs	0.00	0.429
Biological	Coliform (cfu/ml)	PFcs	74.78	0.000
		BF + AC	63.35	0.000
		CF	44.35	0.000

**Table 11:** CWF selection guide for high turbid water (HTW).

Parameters		CWFs	Percentage Correction	Ratio of Leachate to actual of Raw Water
Physico-chemical	Turbidity (NTU)	BF + AC	99.40	0.000
		CF	78.50	0.000
		PFcs	74.27	0.000
	E.C (μS/cm)	PFcs	0.00	0.059
		BF + AC	0.00	1.344
		CF	0.00	1.433
	Total Hardness (mg/l)	CF	0.00	0.688
		PFcs	0.00	0.813
		BF + AC	0.00	1.250
	TDS (mg/l)	PFcs	0.00	0.065
		BF + AC	0.00	1.339
		CF	0.00	1.433
Chemical	pH	BF + AC	0.00	0.149
		CF	0.00	0.132
		PFcs	0.00	0.104
	Fe^2+^ (mg/l)	BF + AC	97.05	0.000
		CF	74.26	0.000
		PFcs	48.82	0.000
	Mn^2+^ (mg/l)	BF + AC	98.49	0.000
		CF	60.14	0.000
		PFcs	54.09	0.000
Biological	Coliform (cfu/ml)	BF + AC	76.97	0.000
		CF	28.65	0.000
		PFcs	2.81	0.000

The effectiveness of a selected ceramic water filter by any user should be informed by the characteristics of the water to be filtered. Therefore, prior knowledge of the capacity of a CWF to be purchased is crucial in its usage. It is thus necessary for, not only manufacturers but government agencies responsible for protecting consumers by ensuring standardization to make available the standard and buying guide for consumers.

It is worth noting that the CWFs used in this study were washed thoroughly before filtration. The ratio of physicochemical, chemical and biological parameter of the leachate (obtained using double distilled deionized water) to those of the raw unfiltered water are as shown in Table 9, Table 10, Table 11 This may also be used as a secondary guide for selection when the percentage correction of the CWFs for any parameter are equal. Some filter materials leach metals and increase the turbidity of the filtrates so it is always better to allow for prefiltration before filtration of the water for drinking.

### 3.5 Water quality index-based guide for selection of CWFs

The selection of CWFs by consumers may also be with respect to the quality of filtered water from different sources using any given CWF. The Water Quality Index (WQI) data in Table 12 provides the WQI values of filtered water from Tap Water (pipe-borne), and surface waters which have low and more visual turbidity respectively (LTW and HTW). Each of the CWFs were used to filter water from the various sources, and the WQI values as shown is believed to have the potential be serve as a guide for consumers who use water with characteristics similar to those used in this study to select the right CWF for use in treatment of their water.

**Table 12:** CWF selection guide based on water quality index.

Water sample	CWFs	WQI	Remarks	Description
**TP**	BF + AC	11.78	Excellent	Drinking, irrigation, industrial irrigation
				Domestic, irrigation, industrial
	CF	81.85	Poor	
	PFcs	45.04	Good	
	Unfiltered water	11.68	Excellent	Drinking, irrigation, industrial
**LTW**	BF + AC	126.38	Very poor	Restricted uses for irrigation
	CF	455.53	Unfit	Proper treatment required before use
	PFcs	745.69		Proper treatment required before use
	Unfiltered water	746.75	Unfit	Proper treatment required before use
**HTW**	BF + AC	37.99	Good	Domestic, irrigation, industrial
	CF	572.69	Unfit	Proper treatment required before use
	PFcs	863.39	Unfit	Proper treatment required before use
	Unfiltered water	2288.14	Unfit	Proper treatment required before use

The WQI scores of the samples of raw water and filtered water ranged from 11.68 to 2288.14 and 11.78–863.39, respectively. WQI of TW was 11.68 (excellent) (Table 12) but after filtration with selected CWFs (BF + AC, PFcs and CF), the filters gave the following WQI values 11.78 (excellent), 45.04 (good) and 81.85 (poor) respectively. This implies that for the used CWFs in treatment of TW, there was a little increase in WQI for BF + AC (9.9), but a significant increase for CF (70.17) and PFcs (33.36) with p-value less than 0.05, suggesting the introduction of leachable materials into the treated water. This could be attributed to the increment in physicochemical parameters such as turbidity, pH, EC and total hardness.

WQI score of 746.75 for LTW was reduced after treatment with CWFs (BF + AC, CF and PFcs) to 126.38, 455.53 and 745.69 respectively (Table 12). Although, there was significant decrease in WQI value of raw water sample by the selected CWFs since p-value <0.05. This indicates that the selected CWFs decreased some physicochemical parameters (turbidity as indicated in Table 3) of the water sample. However, correction of the WQI were 83.08%, 38.99% and 0.14% for BF + AC, CF and PFcs respectively. BF + AC displayed comparatively high correction of WQI followed by CF and PFcs.

WQI score of HTW raw water was 2288.14. For the filtered water, BF + AC reduced it to 37.99, CF to 572.69, and PFcs to 863.39 (Table 12). This implies BF + AC decreased the WQI by 98.34%, CF by 74.97% and PFcs by 62.27% suggesting that the selected CWFs were effective in the correction of physicochemical characteristics. Furthermore, the treatment capacities of CWFs in physicochemical correction is in the order BF + AC > CF > PFcs, this implies BF + AC is more effective compared to CF and PFcs. In addition, the CWFs showed high WQI correction as the physicochemical characteristics of the source water increases. Therefore, the greater the contaminant, the more effective the CWFs. However, BF + AC was effective for the treatment of surface water samples while PFcs and CF were effective for the treatment of tap water. This information is vital as a guide to users of CWFs.

## 4 Conclusion

The efficiency of the three commercially available CWFs (BF + AC, CF and PFcs) in terms of characteristics, metal removability, physicochemical correction and microbial removability were studied. Within the limits of the study, it was evident that the CWFs exhibited very good overall water treatment efficiencies. The CWFs improved physicochemical characteristics of water samples, and the efficiency of the CWF materials were generally in the order BF + AC > CF > PFcs. In addition, the CWFs showed ability to reduce divalent metal (Fe^2+^ and Mn^2+^) ions concentration from raw water.

The metal removability of the CWFs were in the order BF + AC > CF > PFcs, and the CWFs displayed varied capacity for total coliform reduction in raw water sample, the CWFs’ ability was noted to be in the order CF > BF + AC > PFcs for surface water and PFcs > CF > BF + AC for the tap water. In conclusion, CWFs displayed significant correction capacities.

Finally, the results of the study divulged that, the physicochemical and biological parameters and water quality index of the filtrates using any given CWF, have the potential to be used as standard measures for commercially available CWFs. In addition, it was shown that the data is appropriate for use as consumer CWF selection guide.

## Author contribution statement

Ohene B. Apea, Edem Bennet Akorley: Conceived and designed the experiments; Performed the experiments; Analysed and interpreted the data; Contributed reagents, materials, analysis tools or data; Wrote the paper. Emmanuel O. Oyelude: Analysed and interpreted the data; Contributed reagents, materials, analysis tools or data. Boateng Ampadu: Contributed reagents, materials, analysis tools or data.

## Data availability statement

Data will be made available on request.

## Funding

This research did not receive any funding. All financial commitments were borne by the authors in full.

## Declaration of competing interest

The authors declare that they have no known competing financial interests or personal relationships that could have appeared to influence the work reported in this paper.

## Appendix A Supplementary data

Authorship-Change-form-2023.pdf Authorship-Change-form-2023.pdf
