# Supplementary material for: Chemical analysis and filtration efficiency of ceramic point-of-use water filters
Source: Heliyon. 2023 Jul 17;9(7):e18343. doi: 10.1016/j.heliyon.2023.e18343 (PMC10393750; doi:10.1016/j.heliyon.2023.e18343)
Supplement: Authorship-Change-form-2023.pdf [file mmc1.pdf]

Form to confirm authorship changes for *Heliyon*

This form must be ***signed by all authors*** when there is a change in authorship which includes changes to any of the following items: author name(s), order of the authors, the corresponding author(s), the addition of authors, the removal of authors and changes in affiliation.

By personally signing this note, **all** authors confirm that: I) the changes are in accordance with their scientific contribution, II) they agree with all the changes and III) confirm that the authorship list conforms to the authorship criteria outlined on *Heliyon's* ethics page. IV) it is the responsibility of the corresponding author to get the signature from all co-authors accepting the change. In case of any ethic violation/malpractice in the signature, the corresponding author is accountable. The completed form should be returned along with the final/revised manuscript to proceed further with the manuscript. Manuscripts for which incomplete forms have been submitted will be rejected within 5 working days.

Any disputes on the authorship list and contributions need to be resolved by the involved scientists and *Heliyon* will only proceed with the evaluation of the manuscript once we receive confirmation, through this form, that such an agreement between the authors has been reached.

Manuscript number: HELIYON-D-23-04539R3

Article title: CHEMICAL ANALYSIS AND FILTRATION EFFICIENCY OF CERAMIC POINT-OF-USE WATER FILTERS.

Complete new author list: Ohene B. Apea, Edem B. Akorley, Emmanuel O. Oyelude, Boateng Ampadu

Date: 29/06/23

#	First name	Last name	Order change (Y/N)	Addition / Deletion	Change in Author name (Y/N)	Affiliation Change (Y/N)	Reason for the change	Signature
1	Ohene	Apea	Y		N	N	For ease of read and response to requests by readers	[signature]

2	EDEM	AKORLEY	Y		N	N	For ease of reach and response to request by readers.	[illegible]
3	Emmanuel	Oyelude	Y		N	N	For ease of reach.	[illegible]
4	BOATENG	AMPADU	Y		N	N	Has the database and can easily respond to queries.	[illegible]
5								
